# Muscle Strength and Flexibility in Male Marathon Runners: The Role of Age, Running Speed and Anthropometry

**DOI:** 10.3389/fphys.2019.01301

**Published:** 2019-10-16

**Authors:** Pantelis Theodoros Nikolaidis, Juan Del Coso, Thomas Rosemann, Beat Knechtle

**Affiliations:** ^1^Exercise Physiology Laboratory, Nikaia, Greece; ^2^School of Health and Caring Sciences, University of West Attica, Athens, Greece; ^3^Exercise Physiology Laboratory, Camilo José Cela University, Madrid, Spain; ^4^Institute of Primary Care, University of Zurich, Zurich, Switzerland; ^5^Medbase St. Gallen Am Vadianplatz, St. Gallen, Switzerland

**Keywords:** aging, isometric muscle strength, race speed, range of motion, athlete

## Abstract

Most studies on marathon runners have focused on physiological parameters determining performance, whereas neuromuscular aspects, such as muscle strength and flexibility, have received less attention. Thus, the aim of the present study was to examine the relationship of age, body composition, and running speed with muscle strength and flexibility of recreational marathon runners. Male marathon runners (*n* = 130, age 44.1 ± 8.6 years, height 176 ± 6 cm, body mass 77 ± 9 body mass index 24.7 ± 2.6 kg.m^–2^, and race speed 10.29 ± 1.87 km/h) were separated into eight age groups (<30, 30–35, 55–60, >60 years). Four weeks before competing in a marathon, participants performed the sit-and-reach test (SAR), squat jumps (SJ), and countermovement jumps (CMJ), and four isometric muscle strength tests (right and left handgrip, lifting with knees extended and flexed), providing an index of overall isometric muscle strength in absolute (kg) relative to body mass values (kg.kg^–1^ body mass). Afterward, participants competed and finished the Athens Classic Marathon (2017), and race speed was used as an index of running performance. As an average for the whole sample, SAR was 17.6 ± 8.5 cm, SJ was 24.3 ± 4.2 cm, CMJ was 25.8 ± 4.8 cm, overall isometric muscle strength was 386 ± 59 kg in absolute values and 5.06 ± 0.78 kg/kg of body mass in relative terms. The older age groups had the lowest scores in SJ (*p* < 0.001, η_p_^2^ = 0.298) and CMJ (*p* < 0.001, η_p_^2^ = 0.304), whereas no age-related difference in SAR (*p* = 0.908, η_p_^2^ = 0.022), absolute (*p* = 0.622, η_p_^2^ = 0.042) and relative isometric muscle strength (*p* = 0.435, η_p_^2^ = 0.055) was shown. Race speed correlated moderately with relative isometric strength (*r* = 0.42, *p* < 0.001), but not with the other neuromuscular measures (*r* < 0.13,*p* > 0.130). In summary, age-related differences were shown in jumping ability, but not in flexibility and isometric muscle strength. Although these parameters - except relative strength - did not relate to running speed, they were components of health-related physical fitness. Consequently, coaches and runners should consider exercises that include stretching and strengthening in their weekly program to ensure adequate levels for all components of health-related physical fitness.

## Introduction

The number of annual marathon races and finishers has increased during the last few decades (Vitti et al., 2019). Along with the increase in the number of marathoners, this has raised scientific attention to the physiological needs to complete a marathon race in amateur endurance runners, because they constitute the vast majority of finishers in races held worldwide. In this context, several studies have investigated the physiological profile of marathon runners (Del Coso et al., 2017; Salinero et al., 2017). Regarding physiological characteristics of marathon runners, the interplay of maximal oxygen uptake (VO_2__max_), running velocity at lactate threshold, and running economy have been well studied, and they are traditionally considered as the limiting factors for endurance running performance. However, other aspects such as muscle strength and flexibility have received less attention (Takayama and Nabekura, 2018; Takayama et al., 2018) despite their relevance to the above mentioned limiting factors. Even if flexibility and muscle strength were not direct determinants of running performance in marathoners, they are considered as core components of health-related physical fitness (Milanović et al., 2015), and the characterization of these physical aspects through age might help coaches and runners to improve their training programs for marathon competition.

Despite muscle flexibility and muscle strength have been considered as key factors for running performance because of their effect on running economy (Boullosa et al., 2011; Drew et al., 2011), these variables have been studied in long-distance and marathon runners only in a few studies (Jones, 2002; Trehearn and Buresh, 2009; Brown et al., 2011; Nikolaidis et al., 2018a; Del Coso et al., 2019). For instance, isometric muscle strength, sit-and-reach test (SAR), and countermovement jump (CMJ) were tested in a study in female and male marathon runners, but the focus on this investigation was placed on the effects of α-actinin-3 deficiency in these variables (Del Coso et al., 2019). With regards to muscle flexibility, SAR has been negatively associated with running economy, an index of endurance performance (Jones, 2002; Trehearn and Buresh, 2009; Brown et al., 2011). This association indicated that reduced flexibility (SAR) might be advantageous for endurance performance because it might be an indicator of joint and muscle stiffness, variables positively related to running economy (Butler et al., 2003). An interpretation of this association might be reduced SAR, reflected in stiffer musculotendinous structures during the stretch-shortening cycle, which in turn increased storage and return of elastic energy, and consequently improved running economy (Drew et al., 2011).

The existing literature described above enhanced our knowledge on neuromuscular performance of male marathon runners, however, little information existed so far on the variation of muscle strength and flexibility by age, body composition, and running performance in this race distance. Such variations might have practical applications for fitness trainers and coaches in the context of training and testing of their athletes. In addition to their relevance for sport performance, muscle strength and flexibility as components of health-related physical fitness have been mortality predictors, and their optimal values would contribute to the prevention and treatment of lifestyle diseases (e.g., osteoporosis) (Milanović et al., 2019). From a health-related physical fitness perspective, flexibility was widely evaluated using SAR and muscular fitness, measured by isometric tests (e.g., handgrip and lifting) and jump tests (e.g., squat jump, SJ, and CMJ) (Heyward and Gibson, 2014; American College of Sports Medicine [ACSM], 2018). Moreover, although the beneficial role of endurance running for aerobic capacity has been well known (Milanović et al., 2015; Gomez-Molina et al., 2018), less information exists about the neuromuscular fitness levels of humans engaged in regular endurance training. Furthermore, the age of male marathon runners has shown large variation [e.g., 43 ± 10 years in the Berlin marathon (Nikolaidis et al., 2019), 42 ± 10 years in the New York City marathon (Nikolaidis et al., 2018b)], and it would be interesting to investigate the age-related differences in neuromuscular fitness. Therefore, the aim of the present study was to examine the relationship of age, running performance, and body composition with muscle strength and flexibility of recreational marathon runners. A secondary aim was to create norms of neuromuscular fitness that could be applied as a training tool in the evaluation of male recreational marathon runners.

## Materials and Methods

### Study Design and Participants

A cross-sectional study design was adopted in the present study. Male marathon runners (*n* = 130, age 44.1 ± 8.6 years, height 176 ± 6 cm, body mass 77 ± 9 kg, body mass index 24.7 ± 2.6 kg.m^–2^, and race speed 10.29 ± 1.87 km/h) were separated into eight age groups (<30, *n* = 7; 30–35, *n* = 8; 35–40, *n* = 25; 40–45, *n* = 31; 45–50, *n* = 30; 50–55, *n* = 17; 55–60, *n* = 6; >60 years, *n* = 6) and performed SAR, SJ, CMJ, and four isometric muscle strength tests (right and left handgrip, lifting with extended and bended knees), providing an index of overall isometric muscle strength in absolute (kg) and relative to body mass values (kg.kg^–1^ body mass). In addition, all participants in the present study finished the Athens Classic Marathon (2017) 4 weeks after the exercise testing session and race speed was used as an index of endurance running performance. Participants were recruited mostly from Athens through advertisements in social media and local sport clubs, and provided written informed consent after having been enlightened about potential risks and benefits of the study. This study has been approved by the Institutional Review Board of the Exercise Physiology Laboratory, Nikaia, Greece, and has been assigned to the ethical approval number EPL2017/7.

### Equipment and Protocols

Chronological age was estimated by a table of decimals of year (accuracy 0.01 years),considering the date (day/month/year) of exercise testing session and birthday (Ross and Marfell-Jones, 1991). A digital weighting scale (HD-351; Tanita, Arlington Heights, IL, United States) and a stadiometer (SECA, Leicester, United Kingdom) were used to measure body mass and height, respectively, with participants in minimal clothing prior to exercise testing. The ratio of body mass (kg) and height squared (m^2^) estimated body mass index (BMI). Skinfold thickness was measured in ten anatomical sites (cheek, wattle, chest I, triceps, subscapular, abdominal, chest II, suprailiac, thigh, and calf), and their sum was considered to estimate body fat percentage (BF) (Eston and Reilly, 2001). Fat mass (FM) was calculated as “body mass × BF/100,” and fat-free mass (FFM) was “body mass−FM.” Total thigh muscle cross-sectional area (CSA) was estimated from the formula “(4.68 × midthigh circumference in cm)−(2.09 × anterior thigh skinfold in mm)−80.99” (Housh et al., 1995).

Low back and hamstring flexibility was assessed using SAR (Mayorga-Vega et al., 2014), where two trials were performed against a box with the score 15 cm corresponding to the touch of toes. That is, when the participant reached the toes using the fingers, he scored 15 cm. After flexibility, participants performed a 15 min warm-up including cycling and stretching exercises, jumping ability, and isometric muscle strength tests followed. Participants performed two squat jumps (SJ) and two CMJs in counter-balanced order (Aragon-Vargas, 2000); jump height was estimated by the flight time measured with a photocell beams system (Opto-jump, Microgate Engineering, Bolzano, Italy). Both SJs and CMJs were performed with hands stabilized on hips to prevent arm-swing. The two jump tests differed in their starting position, which was with hips and knees flexed in SJ and extended in CMJ. To evaluate isometric muscle strength, four tests were administered - right and left handgrip test, and lifting with extended and flexed knees tests; use of digital handgrip dynamometer (Heyward and Gibson, 2014), and back-and-leg digital dynamometer (Takei, Tokyo, Japan) (Ten Hoor et al., 2016) - and their sum provided an overall score of absolute and relative to body mass muscle strength. In the handgrip test, the grip was adjusted to the palm size and participants were asked to squeeze it in a standing position (Sterkowicz-Przybycien et al., 2019). In the lifting with extended knees, participants stood on the platform of back-and-leg dynamometer and pulled the hand bar across their thighs, whereas the lifting with flexed knees followed the same procedure but with different ankle (i.e. ∼135° instead of 180°) at the knee (Heyward and Gibson, 2014). For the abovementioned exercise tests, 1 min breaks were provided between tests and within trials, and the best of two trials was recorded. During all testing procedures, participants were instructed to perform maximally.

### Statistical Analyses

All statistical analyses were performed by using IBM SPSS v.20.0 (SPSS, Chicago, IL, United States) and GraphPad Prism v. 7.0 (GraphPad Software, San Diego, CA, United States). The data were tested for normality and, thereafter, parametric statistics were used. Mean and standard deviation were calculated for each variable. Differences in SAR, SJ, CMJ, and isometric muscle strength among age groups were examined by one-way analysis of variance (ANOVA) and subsequent Bonferroni *post hoc* tests. The magnitude of the differences was tested by partial eta square, evaluated as small (0.010 < η_p_*^2^* ≤ 0.059), medium (0.059 < η_p_*^2^* ≤ 0.138), and large (η_p_*^2^* > 0.138) (Cohen, 1988). The relationship among variables was examined by Pearson’s product moment correlation coefficient^®^, whose magnitude was interpreted as trivial (*r* < 0.10), small (0.10 ≤ *r* < 0.30), moderate (0.30 ≤ *r* < 0.50), large (0.50 ≤ *r* < 0.70), very large (0.70 ≤ *r* < 0.90), nearly perfect (*r* ≥ 0.90), and perfect (*r* = 1.00) (Batterham and Hopkins, 2006). In addition, 5th, 10th, 25th, 50th, 75th, 90th, and 95th percentile scores were calculated for each neuromuscular parameter. A multiple stepwise regression was run to predict race speed from anthropometric and neuromuscular variables. In addition, a multivariate analysis of covariance (MANCOVA) was performed with muscle strength (absolute and relative overall muscle strength), flexibility and jumping ability (SJ and CMJ) as the dependent variables, age group as the fixed factor, and race speed the covariate. Significance was set at alpha = 0.05, except in the case of MANCOVA, where alpha was corrected to 0.01 (Bonferroni correction) to account for multiple ANOVAs being run.

## Results

**FIGURE 1:**
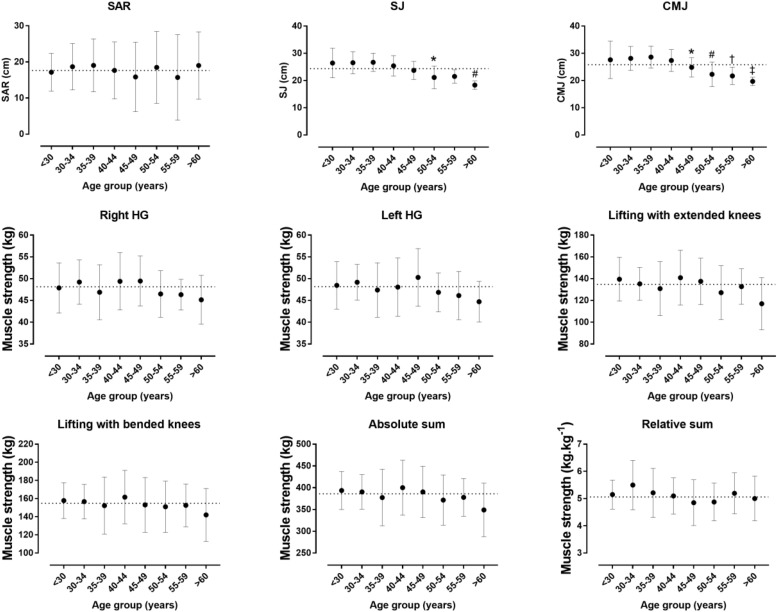
Neuromuscular fitness by age group. SAR, sit-and-reach test; SJ, squat jump; CMJ, countermovement jump; HG, handgrip muscle strength; absolute and relative sum referred to the sum of four measures of isometric muscle strength (right and left HG, lifting with extended and bended knees tests); error bars represented standard deviations; the dashed line showed the mean score of all participants. For SJ: ^∗^, difference of 50–54 age group from <30, 30–34, 35–39, and 40–44 age groups; #, difference of >60 age group from <30, 30–34, 35–39, 40–44, and 45–49 age groups. For CMJ: ^∗^, difference of 45–49 age group from 35–39 age group; #, difference of 50–54 age group from 30–34, 35–39, and 40–44 age groups; †, difference of 55–59 age group from 35–39 age group; ‡, difference of >60 age group from <30, 30–34, 35–39, and 40–44 age groups.

**FIGURE 2:**
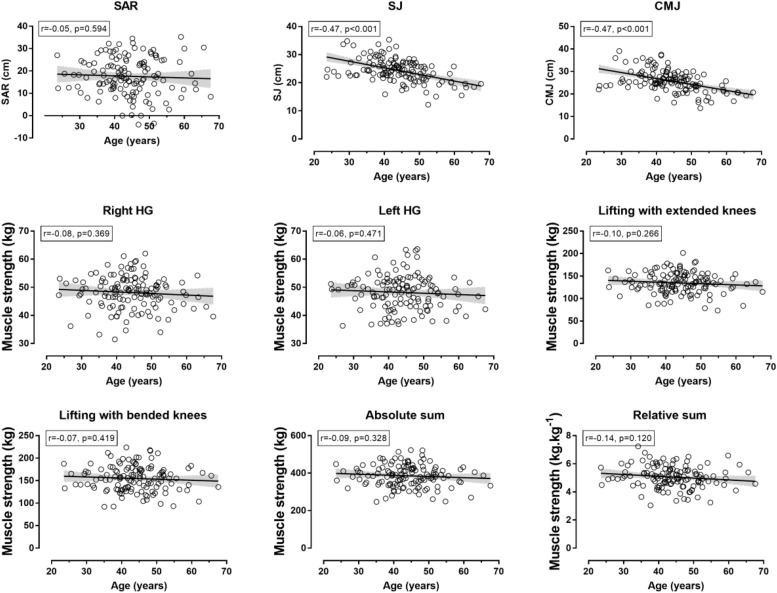
Relationship of neuromuscular fitness with age. SAR, sit-and-reach test; SJ, squat jump; CMJ, countermovement jump; HG, handgrip muscle strength; absolute and relative sum referred to the sum of four measures of isometric muscle strength (right and left HG, lifting with extended and bended knees tests); shadowed areas represented 95% confidence intervals.

Table 1 depicts information about the values in all testing protocols as a whole group, including ranges. Briefly, SAR was 17.6 ± 8.5 cm, SJ was 24.3 ± 4.2 cm, CMJ was 25.8 ± 4.8 cm, absolute strength was 386 ± 59 kg and relative strength was 5.06 ± 0.78 kg/kg of body mass. Percentile norms are presented in Table 2. The older age groups had the lowest scores in SJ (*p* < 0.001, η_p_^2^ = 0.298) and CMJ (*p* < 0.001, η_p_^2^ = 0.304), whereas no age-related difference in SAR (*p* = 0.908, η_p_^2^ = 0.022), absolute (*p* = 0.622, η_p_^2^ = 0.042) and relative isometric strength (*p* = 0.435, η_p_^2^ = 0.055) was shown (Figure 1). Age correlated moderately with SJ (*r* = −0.47, *p* < 0.001) and CMJ (*r* = −0.47, *p* < 0.001), but not with the other neuromuscular measures (*r* < 0.14, *p* > 0.120) (Figure 2). Race speed correlated moderately with relative isometric strength (*r* = 0.42, *p* < 0.001), but not with the other neuromuscular measures (*r* < 0.13, *p* > 0.130).

**TABLE 1:** Descriptive statistics of participants (*n* = 130).

**Parameter**	**Mean**	**SD**	**Range**
Age (years)	44.1	8.6	23.5–67.6
Height (cm)	176.4	5.8	163.2–196.6
Weight (kg)	76.9	9.4	56.2–108.1
BMI (kg.m^–2^)	24.7	2.6	19.1–35.0
BF (%)	17.7	4.1	7.8–27.1
FFM (kg)	63.0	6.1	48.9–79.9
CSA (cm^2^)	141.5	14.1	111.2–189.3
Race speed (km.h^–1^)	10.3	1.9	6.0–15.0
SAR (cm)	17.6	8.5	−4–35.3
Right HG (kg)	48.1	5.9	31.5–62.0
Left HG (kg)	48.2	6.0	36.3–63.6
Sum of and right and left HG (kg)	96.4	11.4	68.6–125.6
Lifting with extended knees (kg)	134.8	23.1	73.5–201.5
Lifting with flexed knees (kg)	154.9	28.3	92.0–224.0
Absolute sum (kg)	386.0	58.5	247.0–523.0
Relative sum (kg.kg^–1^)	5.06	0.78	3.04–7.24
SJ (cm)	24.3	4.2	12.2–35.3
CMJ (cm)	25.8	4.8	13.7–39.1

*BMI, body mass index; BF, body fat percentage; FFM, fat-free mass; CSA, total thigh muscle cross-sectional area; SAR, sit-and-reach test; HG, handgrip; SJ, squat jump; CMJ, countermovement jump. The absolute and relative sum referred to the sum of the four isometric muscle strength tests (right and left handgrip test, lifting with extended and flexed knees tests).*

**TABLE 2:** Percentile values of neuromuscular fitness.

	**Percentile**						
**Parameter**	**5**	**10**	**25**	**50**	**75**	**90**	**95**
SAR (cm)	2.9	6.5	12.0	17.4	24.1	29.2	30.4
Right HG (kg)	38.1	39.7	44.3	48.7	52.3	55.1	57.8
Left HG (kg)	37.8	39.3	43.7	49.1	52.0	54.9	59.8
Sum of and right and left HG (kg)	76.5	81.8	88.2	98.2	103.3	110.5	115.5
Lifting with extended knees (kg)	90.1	106.0	121.1	135.0	152.0	163.3	172.3
Lifting with flexed knees (kg)	105.9	120.8	133.3	155.5	174.4	192.0	203.7
Absolute sum (kg)	282.7	307.5	344.2	387.0	425.7	461.5	479.7
Relative sum (kg)	3.62	4.09	4.60	5.05	5.55	6.10	6.37
SJ (cm)	17.0	19.1	21.7	24.5	27.1	29.6	32.5
CMJ (cm)	17.2	20.1	23.2	25.5	28.5	32.7	35.0

*BMI, body mass index; SAR, sit-and-reach test; HG, handgrip; SJ, squat jump; CMJ, countermovement jump. The absolute and relative sum referred to the sum of the four isometric muscle strength tests (right and left handgrip test, lifting with extended and flexed knees tests).*

**FIGURE 3:**
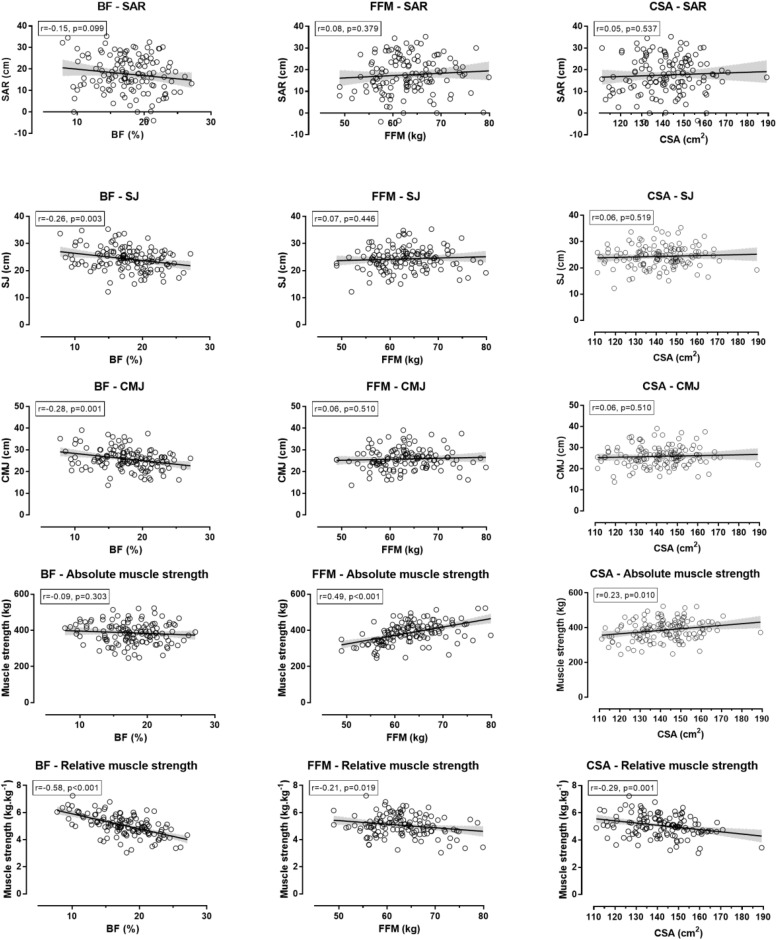
Relationship of flexibility, jumping ability and isometric muscle strength with body fat percentage, fat-free mass and total thigh muscle cross-sectional area. Muscle strength referred to the sum of four measures (**right** and **left** handgrip test, back test, back-and-leg test); BF, body fat percentage; FFM, fat-free mass; CSA, total thigh muscle cross-sectional area; SAR, sit-and-reach test; SJ, squat jump; CMJ, countermovement jump; shadowed areas represented 95% confidence intervals.

Sit-and-reach test was not related to BF, FFM, and CSA (Figure 3). SJ and CMJ were negatively related with small magnitude to BF, but not to FFM and CSA. The absolute muscle strength was related directly to FFM (moderate magnitude) and CSA (small magnitude), but not to BF. The relative muscle strength was negatively related to BF (large magnitude), FFM, and CSA (small magnitude).

The results of the multiple stepwise regression showed that race speed could be predicted by BF, age, BMI, and CMJ (*R*^2^ = 0.54) (Table 3). According to MANCOVA, there was a statistically significant difference of medium magnitude in neuromuscular characteristics based on participants’ age adjusted for race speed (*F*_35__,__482_ = 2.134, *p* < 0.001, Wilk’s Λ = 0.545, η_p_^2^ = 0.114). There was a statistically significant effect of age group on SJ (*F*_7__,__118_ = 6.196, *p* < 0.001, η_p_^2^ = 0.269) and CMJ (*F*_7__,__118_ = 6.448, *p* < 0.001, η_p_^2^ = 0.277), but not on SAR (*F*_7__,__118_ = 0.277, *p* = 0.962, η_p_^2^ = 0.016), absolute (*F*_7__,__118_ = 0.739, *p* = 0.640, η_p_^2^ = 0.042), and relative muscle strength (*F*_7__,__118_ = 0.382, *p* = 0.911, η_p_^2^ = 0.022).

**TABLE 3:** Model summary of stepwise regression to predict race speed from anthropometric and neuromuscular characteristics.

**Model**	**Variables**	***R***	***R*^2^**	**SEE**	**Δ*R*^2^**
1	BF^a^ (−0.644)^b^	0.644	0.415	1.44	0.415
2	BF (−0.603), age^a^ (−0.229)	0.683	0.466	1.38	0.051
3	BF (−0.403), age (−0.224), BMI^a^ (−0.285)	0.712	0.507	1.33	0.041
4	BF (−0.446), age (−0.307), BMI (−0.277), CMJ^a^ (−0.199)	0.733	0.537	1.29	0.030

*BF, body fat percentage; BMI, body mass index; CMJ, countermovement jump; *R*, coefficient of multiple correlation; *R*^2^, coefficient of determination; SEE, standard error of the estimate; Δ*R*^2^, *R*^2^ change resulting from entering a new variable, ^*a*^ denotes variable entered in each model, ^*b*^ standardized beta value was presented in brackets next to variable.*

## Discussion

The main findings of the present study were that (a) older age groups of recreational male marathon runners had lower SJ and CMJ than their younger counterparts, (b) no difference in SAR and (absolute and relative) isometric muscle strength was observed among age groups, (c) race speed correlated with relative isometric muscle strength, but not with the other neuromuscular measures, and (d) BF was negatively related to SJ, CMJ, and relative muscle strength.

### Comparison Among Age Groups

The lower scores in SJ and CMJ in the older compared to the younger age groups, and the absence of difference in SAR and isometric muscle strength among age groups were in agreement with the correlation analysis that identified negative relationship of SJ and CMJ - and no relationship for SAR and isometric muscle strength - with age. A negative correlation of CMJ with age was previously observed in distance runners (Michaelis et al., 2008; Nikolaidis et al., 2018a). In addition, a comparison of jumping ability, as it was reflected in jumping disciplines of athletics, among age groups showed lower performance in the older age groups than their younger peers (Kundert et al., 2019a, b). These findings together suggested a decline of SJ and CMJ with age, which should be attributed to a decline of muscle fiber type II area and fat-free mass with age (Hawkins et al., 2003). An explanation of the absence of age-related differences for SAR and isometric muscle strength, or the observation of small differences in jump performance might also be a reflection of the relatively low neuromuscular fitness of participants (Nikolaidis et al., 2016).

### Relationship Between Race Speed and Neuromuscular Fitness

The moderate correlation of race speed with relative isometric muscle strength indicated that a fast marathon runner would be characterized by high muscle strength when the role of body mass was partitioned out. It has been observed previously that fast marathon runners did not differ from their slower counterparts with regards to absolute isometric muscle strength, however, when their body mass was considered, the former runners had higher relative muscle strength than the latter ones (Salinero et al., 2017). Although the application of force in leg muscles during endurance running is far from its maximal expression, high levels of relative muscle strength might be useful for marathoners in order to reduce the exercise-induced muscle damage level developed during the race (Del Coso et al., 2013) or to increase running economy (Giovanelli et al., 2017). Because muscle strength is a trainable parameter in marathoners with multiple benefits for marathoners, especially in the amateur population, concurrent resistance and endurance training should be implemented in replacement of the traditional vision of “only-endurance” training to increase overall marathon performance (Yamamoto et al., 2008).

### Neuromuscular Fitness and Anthropometric Characteristics

The absence of relationships between SAR and BF, FFM, and CSA was expected, since flexibility has been a musculoskeletal attribute rather than a correlate of body composition. The negative relationship of SJ and CMJ with BF was explained from the observation that an additional FM consisted extra load that muscle strength of lower limbs should overtake (Gatterer et al., 2013). With regards to isometric muscle strength, the relationship of the absolute overall score with FFM (medium magnitude) and CSA (small magnitude) was in agreement with research demonstrating the association between muscle strength and muscle CSA (Fink et al., 2017). That is, an increased absolute overall muscle strength of participants was related to increased FFM and CSA. Interestingly, this trend was reversed when overall muscle strength was expressed relative to body mass values, since muscle strength depended on both muscular and neurological properties (McKay et al., 2017). This finding highlighted the need to assess and interpret muscle strength values in both absolute and relative to body mass values (Heyward and Gibson, 2014).

### Limitations, Strength and Practical Applications

A limitation of the present study was the assessment methods of neuromuscular fitness; although popular measures of flexibility (SAR), muscle strength (isometric dynamometry), and jumping ability (SJ and CMJ) were used, caution would be needed in the consideration of methodological details to compare the findings with previous research. For instance, 8.8 cm was the SAR score of distance runners in a study, where zero was set at the toes (Jones, 2002), in contrast to the 15 cm set at the toes in the present study. Thus, to have comparable data, 15 cm should be added to the data of Jones (2002). Moreover, it was acknowledged that the existence of age groups with unequal sample sizes (most participants were in the 40–45 age group, and their number was decreasing in the younger and older groups) might be subjected to criticism from a statistical point of view. It should be highlighted that the existence of unequal sample sizes in age groups of marathon runners was ecologically valid, since it was representative of the variation in the participation rates by age group in marathon races. For instance, most male marathon runners were in the 40–44 age group in the New York City Marathon (Nikolaidis et al., 2018b) and in the Berlin marathon race (Nikolaidis et al., 2019).

In addition, the period between exercise testing session and marathon race was ∼4 weeks, and physiological characteristics could change during this period. Actually, there has been evidence that although a 3 months typical endurance running protocol (three 60 min sessions per week) improved aerobic capacity, no change in SAR, SJ, and CMJ was observed (Milanović et al., 2015). Similarly, a 2 months endurance running protocol (three ∼60 min sessions per week) improved aerobic capacity, but not SJ and CMJ (Gomez-Molina et al., 2018). Therefore, it might be assumed that neuromuscular characteristics of participants in the present study would be similar both in the exercise testing session and in the date of race.

On the other hand, the measurement of several strength variables is one of the novelties of this investigation, since it was the first study - to the best of our knowledge - presenting data on a complete battery of neuromuscular fitness tests in a large sample of marathon runners through a large age range. For practical applications, coaches and fitness trainers working with marathon runners might benefit from the novel data presented during the training and testing of their athletes.

## Conclusion

In summary, age-related differences were shown in jumping ability, but not in flexibility and isometric muscle strength. Although these parameters - except relative strength - did not relate to marathon performance, they were components of health-related physical fitness. Consequently, coaches and runners should consider exercises including stretching and strengthening in their weekly program to ensure adequate levels for all components of health-related physical fitness.

## Data Availability Statement

The datasets generated for this study are available on request to the corresponding author.

## Ethics Statement

The study has been approved by the Institutional Review Board of the Exercise Physiology Laboratory, Nikaia, Greece, and has been assigned to the ethical approval number EPL2017/7.

## Author Contributions

PN performed the experiments and drafted the manuscript. JD, TR, and BK helped in drafting the final manuscript.

## Conflict of Interest

The authors declare that the research was conducted in the absence of any commercial or financial relationships that could be construed as a potential conflict of interest.
